# Acute cholangitis in an old patient with Crigler-Najjar syndrome type II - a case report

**DOI:** 10.1186/s12876-016-0449-9

**Published:** 2016-03-11

**Authors:** Samuel Raimundo Fernandes, Carlos Miguel Moura, Beatriz Rodrigues, Luís Araújo Correia, Helena Cortez-Pinto, José Velosa

**Affiliations:** Serviço de Gastrenterologia e Hepatologia, Hospital de Santa Maria, Centro Hospitalar Lisboa Norte, Avenida Professor Egas Moniz, Lisboa, 1649-035 Portugal; Avenida Tomás Fonseca n° 36, 13, Lisbon, B 1600-275 Portugal

**Keywords:** Crigler Najjar, Hyperbilirubinemia, Acute cholangitis, Choledocolitiasis, Uridine diphosphate-glucuronosyl-transferase deficiency

## Abstract

**Background:**

Crigler-Najjar syndrome (CN) is a very rare genetic disorder characterized by an inability to conjugate bilirubin. Contrary to CN type I, patients with CN II exhibit residual capacity to conjugate bilirubin and may present a normal life expectancy.

**Case presentation:**

We report an unusual late diagnosis of CN type II in an 80-year-old female admitted with severe acute cholangitis. While the patient present typical clinical and radiologic signs of bile duct obstruction and cholangitis, her blood analysis showed severe unconjugated hyperbilirubinemia. Endoscopic retrograde cholangiopancreatography confirmed the diagnosis and allowed therapeutic intervention. The anatomopathologic examination of her gallbladder following cholecystectomy showed signs of chronic cholecystitis.

**Conclusion:**

The risk of gallstone disease may be increased in patients with CN syndrome. While unusual, we alert to this curious and potential life-threatening presentation.

## Background

Crigler-Najjar syndrome (CN) is a rare autosomal recessive disorder with an incidence estimated at 0.6 patients per million [1]. It is characterized by impaired activity of the hepatic enzyme uridine diphosphate-glucuronosyl-transferase (UDP-GT). In CN type I there is a complete absence of UDP-GT and these patients present with severe accumulation of unconjugated bilirubin in basal ganglia and cerebellum, leading to irreversible brain damage in the first years of life [1]. Patients with CN type II exhibit severe deficiency of UDP-GT enzyme activity (below 30 %). It is a less severe form of the disease, usually without neurological damage, although patients may still require phototherapy and early liver transplantation to improve quality of life. Mutations in UDP-GT have been associated with an increased risk of gallstone formation [2–8]. The underlying mechanism is believed to involve increased levels of unconjugated bilirubin in the bile leading to increased formation of calcium-bilirubinate compounds [9]. In 1983, Trotman described the first report of acute cholangitis secondary to bile duct obstruction in a young patient with CN type II [10]. In this paper, we report the second case of acute cholangitis in a patient with CN type II. In addition, we discuss this uncommon presentation in an elderly patient, and review the available literature on this disease.

## Case presentation

**Fig. 1:**
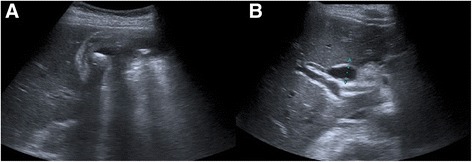
Abdominal ultrasound showing gallbladder wall thickening, pericholecystic fluid and cholelithiasis (**a**). The common bile duct is dilated (**b**)

**Fig. 2:**
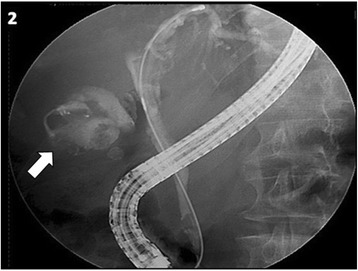
Endoscopic retrograde cholangiopancreatography showing multiple stones in the gallbladder (arrow)

An 80-year-old female patient was admitted to Santa Maria Hospital’s emergency department with acute right upper quadrant abdominal pain, fever, chills and vomiting. Her medical history was relevant for CN type II, systemic hypertension and type II diabetes *mellitus*. Although she presented persistent jaundice since birth, the diagnosis of CN was only made in adulthood, based only on clinical grounds. She had never required treatment for CN. Seven years earlier, she had been admitted to a different institution with cholecystitis and gallstone pancreatitis. She underwent therapeutic endoscopic retrograde cholangiopancreatography (ERCP) with sphincterotomy. The patient declined cholecystectomy for personal reasons. A laboratory evaluation requested by her general practitioner 5 years earlier showed normal aspartate aminotransferase (AST) – 24 U/L (normal range < 34 U/L), gama-glutamyl-transpeptidase (GGT) - 30 U/L (normal range < 32 U/L), prothrombin time – 11.6 s (normal range <11.8 s), markedly increased total bilirubin - 11.7 mg/dl (normal range <1.0 mg/dl), and only slightly elevated direct bilirubin - 0.37 mg/dl (normal range <0.35 mg/dl). At admission, physical examination showed fever (39 °C), severe icterus and right upper quadrant tenderness. Laboratory tests revealed signs of systemic inflammation (leukocytosis, elevated C-reactive protein) and a cytocholestatic pattern with unusual elevation in total bilirubin, but only a slight elevation of the direct fraction. There was no evidence of hemolysis (Table 1). Abdominal ultrasound demonstrated distention of the gallbladder with marked wall thickening (10 mm), pericholecystic fluid, positive sonographic Murphy's sign and cholelithiasis. The intrahepatic and common bile ducts were dilated (11 mm) and there was an echogenic material within the common bile duct suggestive of gallstones (Fig. 1). This was compatible with acute obstructive cholangitis with cholecystitis. After starting broad spectrum antibiotics, the patient underwent emergency ERCP which revealed multiple gallbladder and common bile duct stones. Following exploration with basket and balloon, sludge and pus were observed coming out from the papilla, thus confirming cholangitis (Fig. 2). The patient evolved uneventfully. By the 7^th^ day of admission there was a clear improvement in cholestasis (Table 2). She was discharged after 10 days of hospitalization. Four weeks later, the patient underwent elective laparoscopic cholecystectomy. The surgical specimen measured 7 × 3.5 cm. The gallbladder wall was moderately thickened. The outer surface was partially rough, yellow-gray with multiple hemorrhagic foci. The lumen exhibited multiple ovoid yellow-brown stones, the largest measuring 15 mm. Rokitansky–Aschoff sinuses could also be seen. These findings were compatible with chronic calculous cholecystitis.

**Table 1:** Laboratory evaluation at admission. Inflammatory markers and acute renal failure is present. In addition a cytocholestatic pattern is apparent with unusual disproportion in total to direct bilirubin

Item	Value	Normal values
Hemoglobin (g/dL)	12.0	12.0–15.3
Leucocytes (x 10^9^/L)	12.3	4.0–10.0
Neutrophils (x 10^9^/L)	11.2	1.5–7.5
Platelets (x 10^9^/L)	93	150–400
Creatinine (mg/dl)	2.0	0.6–1.3
CRP (mg/dl)	11.0	<0.5
LDH (UI/ml)	250	65–387
AST (U/L)	1085	(<34)
ALT (U/L)	934	(12–78)
GGT (U/L)	330	(<38)
Total bilirubin (mg/dL)	19.4	(<1.0)
Direct bilirubin (mg/dL)	2.71	(<0.3)
Haptoglobin (mg/dL)	234	(30–200)

**Table 2:** Laboratory results at discharge. There is a clear improvement in the liver panel

Item	Value	Normal values
AST (U/L)	21	(<34)
GGT (U/L)	116	(<38)
Total bilirubin (mg/dL)	15.6	(<1.0)
Direct bilirubin (mg/dL)	1.1	(<0.3)

## Discussion

CN type II, unlike type I, is a potentially benign disease. Patients with this condition usually have serum bilirubin levels ranging between 10 and 20 mg/dL (175 and 350 μmol/l) and seldom develop kernicterus, although neurological dysfunction may occur if hyperbilirubinemia is exacerbated by fasting, drugs or infectious diseases [11]. There have been several published reports of increased incidence of gallstones in patients with UDP-GT mutations [2–5, 12]. In a series with 20 patients with CN type I followed over a 16-year period, half developed cholelithiasis and required elective cholecystectomy [12]. Gallstones were detected on cholecystography in 4 of 53 patients with Gilbert’s disease by Foulk et al. [3] and required cholecystectomy in 2 of 55 patients studied by Powell et al. [2] In a series of cholecystectomy patients, Gilbert’s disease was reported in 7.5 % of male patients [4]. Several explanations for this occurrence have been suggested. One theory relates to the composition of bile which is markedly abnormal in patients with UDP-GT mutations [9, 13]. Due to the inability to adequately conjugate bilirubin, the bile of patients with UDP-GT mutations exhibits abnormal amounts of bilirubin mono and diglucuronides. These compounds are more prone to deconjugation by bacterial glucuronidases. Unconjugated bilirubin combines with bile calcium, leading to the formation of pigmented gallstones [9, 13]. This however, may prove to be insufficient as a physiopathological explanation, since most reports of gallstones in patients with UDP-GT mutations describe an additional factor such as cystic fibrosis, sickle cell disease or spherocytosis [6–8]. Nevertheless, despite an elevated risk for gallstone disease, we have only found one report of cholangitis in a patient with CN type II [10]. Differentiation between Gilbert’s disease and CN type I and II is important since treatment and prognosis are different between these entities. Although the diagnosis is usually made on clinical grounds, response to phenobarbital (decrease by at least 25 % in CN type II), bile analysis, measurement of UDP-GT on liver tissue and genetic testing can be used to confirm the diagnosis [14–17]. Interestingly, patients with atypical presentations preventing a clear differentiation between type I and II have been described, raising awareness for possible phenotypes with incomplete penetration [18, 19]. Although generally unnecessary, treatment options for patients with CN type II include phototherapy [1], plasmapheresis [20], enzyme induction with phenobarbital [4] and liver transplantation [1, 21]. Phototherapy effectively decreased unconjugated bilirubin by converting it to a water-soluble photoisomer that can be excreted in the bile. This treatment is only temporary as skin thickness and body surface area increase with age, reducing its efficiency. Additionally, phototherapy represents a heavy burden for the patient, requiring 10 to 12 h/day of treatment. The average life expectancy for patients having type I syndrome is around 30 years [21]. Even though the prognosis of CN type II is usually good and is compatible with a normal life span, repeated episodes of kernicterus may culminate in permanent neurological damage [22, 23]. The present case seemed of great interest due to the rarity of the situation, and also because of the advanced age of presentation, with the patient only presenting this frequent complication at this old age. Aside from the episode of acute gallstone pancreatitis, 7 years earlier, no other significant complications of the prolonged hyperbilirubinemia were seen in her lifetime.

## Conclusion

CN patients are at an increased risk for gallstones and associated complications. This is the second report of cholangitis in a patient with CN, nevertheless, as cholangitis is potentially fatal if unrecognized and left untreated, clinicians should be aware of this possibility and endeavor to exclude it early. As these patients are permanently jaundiced, we cannot rely solely on this sign to diagnose cholangitis. In addition, the literature suggests that pigmented gallstones may follow in the long-term of mild cases of CN.

## Consent

Written informed consent was obtained from the patient for publication of this Case report and any accompanying images. A copy of the written consent is available for review by the Editor of this journal.

## Abbreviations

CN: Crigler Najjar
ERCP: cholangiopancreatography
UDP-GT: uridine diphosphate-glucuronosyl-transferase
